# Long-Term Hepatitis B Surface Antigen Profile and Seroclearance Following Antiviral Treatment: A Single-Center, Real-World Cohort Study

**DOI:** 10.3390/biomedicines11112966

**Published:** 2023-11-03

**Authors:** Chih-Wen Huang, Chen-Ta Yang, Pei-Yuan Su, Yang-Yuan Chen, Siou-Ping Huang, Hsu-Heng Yen

**Affiliations:** 1 Division of Gastroenterology, Department of Internal Medicine, Changhua Christian Hospital, Changhua 500, Taiwan; 2 Department of Post-Baccalaureate Medicine, College of Medicine, National Chung Hsing University, Taichung 402, Taiwan

**Keywords:** chronic hepatitis B, Entecavir, Tenofovir alafenamide, finite therapy, clinical relapse

## Abstract

Hepatitis B surface antigen (HBsAg) seroclearance, an indicator of recovery from hepatitis B virus (HBV) infection, is uncommon in long-term nucleos(t)ide analog (NUC) therapy. We compared the incidence of HBsAg seroclearance in patients with and without NUC discontinuation to identify predictors of HBsAg seroclearance. This retrospective study enrolled adult patients with a chronic HBV infection followed for ≥12 months after NUC discontinuation (finite group) and those treated with NUCs for >3 years (non-finite group). Demographic, clinical, and laboratory data were analyzed. The study cohort included 978 patients, including 509 and 469 patients in the finite and non-finite groups, respectively. Cumulative HBsAg seroclearance incidence was significantly higher in the finite group than in the non-finite group (*p* = 0.006). The 5- and 10-year cumulative HBsAg seroclearance incidence were 6.6% and 18.9% in the finite group and 3% and 14.6% in the non-finite group, respectively. The likelihood of HBsAg seroclearance was higher in those with end of treatment (EOT) HBsAg levels of <100 IU/mL and in those without clinical relapse (CR). The cumulative 3-year CR incidence was 16.8%. The incidence of liver decompensation and hepatocellular carcinoma were 4.1 and 0.4 per 1000 person-years, respectively. The hepatocellular carcinoma incidence did not significantly differ between the finite and non-finite groups (*p* = 0.941). In conclusion, higher HBsAg seroclearance incidence in patients receiving finite therapy, and the increased likelihood of HBsAg seroclearance in those with EOT HBsAg levels of <100 IU/mL and in those without CR should be considered during decision-making of treatment options.

## 1. Introduction

The prevalence of hepatitis B virus (HBV) infection has gradually declined with the introduction of the HBV vaccine. However, chronic HBV infection (CHB) continues to play a key role in progression to cirrhosis and hepatocellular carcinoma (HCC). Nucleos(t)ide analogs (NUCs), such as entecavir, tenofovir disoproxil fumarate (TDF), and tenofovir alafenamide (TAF), which provide excellent suppression of viral replication and a high barrier to viral resistance, are recommended as first-line therapy for CHB [1]. Despite its ability to suppress HBV replication and reduce hepatocellular inflammation, NUC therapy cannot completely eradicate HBV [2,3,4]. The ultimate goal for a functional HBV cure is rarely achieved, despite long-term NUC therapy [5]. In two cohort studies investigating outcomes following long-term entecavir or TDF treatment, the 8- and 10-year cumulative HBsAg seroclearance incidence rates were 1.69% and 2.11%, respectively [6,7]. Furthermore, recent studies have reported higher HBsAg seroclearance rates in patients who discontinued NUC therapy compared to those who continued NUC therapy. However, CHB might relapse and lead to hepatitis flare, hepatic decompensation, and even death after NUC therapy discontinuation [8,9,10,11,12].

Various NUC discontinuation guidelines have been proposed to address side effects associated with long-term NUC use, financial concerns, and patient compliance while aiming to achieve functional cure. Discontinuing NUC therapy after HBV e antigen (HBeAg) loss and consolidation therapy for >12 months are suggested in HBeAg-positive patients. The Asian Pacific Association for the Study of the Liver (APASL) suggests NUC therapy discontinuation after treatment for at least two years in HBeAg-negative patients with CHB who have undetectable HBV DNA documented on three separate occasions six months apart, whereas guidelines from Western countries recommend infinite duration of antiviral therapy [1,13,14]. Additionally, information is limited regarding the incidence of HBsAg seroclearance and predictors of HBsAg seroclearance after the discontinuation of NUC therapy. Furthermore, the association of TAF, a recently approved NUC as a first-line treatment option for CHB [3], with HBsAg seroclearance remains unclear.

Therefore, we conducted a real-world study to examine the incidence of HBsAg seroclearance in patients with and without NUC therapy discontinuation based on the APASL guidelines and the Taiwanese National Health Insurance reimbursement requirements. In addition, we aimed to determine the best candidate patients for discontinuing NUC therapy to achieve HBsAg seroclearance. We hoped to gain a deeper understanding of the benefits and downsides of finite therapy in accordance with the APASL guidelines and the Taiwanese National Health Insurance reimbursement criteria as well as the incidence of HBsAg seroclearance in patients treated with specific NUCs.

Therefore, we compared the CR rates between TAF and entecavir therapies 48 weeks after cessation of their anti-viral therapy.

## 2. Materials and Methods

### 2.1. Materials

#### 2.1.1. Patients

This was a retrospective, single-center, real-world study including all patients with CHB who received NUC therapy at Changhua Christian Hospital between 1 January 2008 and 23 April 2022. The study population included patients without HCC, cirrhosis, alcoholic liver disease, hepatitis C virus infection, autoimmune hepatitis, and immunosuppressive treatment before initiating NUC therapy. All patients received entecavir, TDF, or TAF for at least two years in Changhua Christian Hospital in accordance with the reimbursement guidelines of the Taiwanese National Health Insurance. In Taiwan, NUC therapy was reimbursed in patients meeting the following criteria: HBsAg positivity for more than six months, 2–5-fold increase in alanine aminotransferase (ALT) levels from the upper normal limit, and HBV DNA levels of >20,000 IU/mL in HBeAg-positive patients; and HBsAg positivity for more than six months, 2–5-fold increase in ALT levels from the upper normal limit, and HBV DNA levels of >2000 IU/mL in HBeAg-negative patients. NUC therapy was discontinued in HBeAg-positive patients after three years of antiviral therapy. NUC therapy was discontinued in HBeAg-negative patients after three years of antiviral therapy or with undetectable serum HBV DNA levels on three independent occasions more than six months apart.

In the present study, these patients were categorized as the finite group following the stopping rule of insurance reimbursement guidelines. In contrast, patients who continued NUC therapy during the study period were categorized as the non-finite group.

During the study period, there were changes in Taiwan’s National Health Insurance reimbursement regulations, allowing for longer periods of medication coverage. As a result, most of our non-finite group patients came from the later study period.

**Figure 1:**
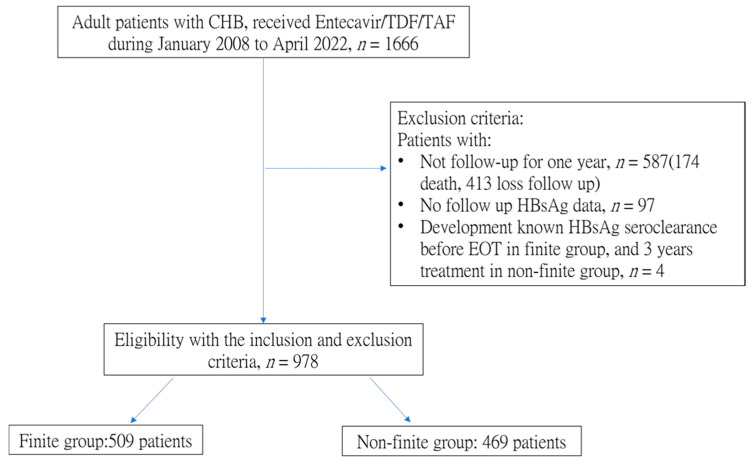
Patient disposition during the study. CHB, chronic hepatitis B; TAF, tenofovir alafenamide; TDF, tenofovir disoproxil fumarate.

Patients who were not followed for more than one year, had no follow-up HBsAg data, or developed HBsAg seroclearance before the end of treatment (EOT) in the finite group and before 3 years of treatment in the non-finite group were excluded from the study. Figure 1 shows the study flow chart.

#### 2.1.2. Follow-Up and Clinical Relapse

For patients in the finite group, ALT and HBsAg levels were monitored every six months following NUC therapy discontinuation, until the final visit, or until HBsAg seroclearance. After three years of treatment, the patients in the non-finite group were followed every six months until the final visit or HBsAg seroclearance. Serum HBV DNA levels were determined in cases where the ALT level was more than 2-fold over the upper normal limit (41 IU/mL). Clinical relapse (CR) was defined as a 2-fold increase in ALT levels and HBV DNA levels of >2000 IU/mL.

The incidence of HBsAg seroclearance was compared between the finite and non-finite groups, and the Cox regression model was used to study the determinants of HBsAg seroclearance.

The study was conducted in accordance with the Declaration of Helsinki and approved by the Institutional Review Board of Changhua Christian Hospital (protocol code: 230509).

### 2.2. Statistical Analyses

Data were expressed as numbers (%), medians (interquartile range), or mean ± standard deviation. Categorical variables were compared using the χ2 test, and continuous variables were compared using Student’s *t* or the Mann–Whitney *U* test, as appropriate. The Kaplan–Meier method with the log-rank test was used to analyze and compare the cumulative incidence rates of HBsAg seroclearance. The study entry date was the day of EOT in the finite group and the day after three years of treatment in the non-finite group. Time at risk as a continuous variable was measured from the entry date until HBsAg seroclearance or the date of last available HBsAg data, whichever came first. R software for Windows (version 4.0.5) and the “survival” (https://cran.r-project.org/web/packages/survival/index.html, accessed on 1 August 2023) and “survminer” (https://cran.r-project.org/web/packages/survminer/index.html, accessed on 1 August 2023) packages were used to generate cumulative incidence plots. Cox proportional hazards regression models were used to conduct univariate and multivariate analyses for HBsAg seroclearance. In the univariate analysis, variables that showed significance (*p* < 0.05) were included in the multivariate analysis. The multivariate analysis utilized the forward method to identify independent factors, and variables with a *p*-value of less than 0.05 were retained in the models. All statistical analyses were performed using IBM SPSS Statistics for Windows version 20 (IBM, Armonk, NY, USA). A *p* value of <0.05 was considered as statistically significant.

## 3. Results

### 3.1. General Characteristics of the Study Population

The study cohort included 978 patients who fulfilled the inclusion and exclusion criteria. Table 1 summarizes the clinical characteristics of the study population, including 509 and 469 patients in the finite and non-finite groups, respectively. The two groups did not significantly differ in sex distribution, body mass index, baseline ALT levels, total bilirubin levels at baseline, HBsAg levels of EOT in finite group, and of 3 years after treatment in non-finite group, or serum HBV DNA levels at baseline. The patients in the finite group were significantly older than those in the non-finite group (mean age, 49.1 ± 13.3 vs. 46.9 ± 12.5 years). The duration of follow-up and NUC therapy were significantly shorter in the finite group than in the non-finite group (45.4 [21.1–104.1] vs. 63.4 [42–99.3] and 35.7 [24.1–36.5] vs. 46.3 [38.5–70.1] months, respectively).

### 3.2. Comparison of Cumulative Incidence Rates between the Finite and Non-Finite Groups

**Figure 2:**
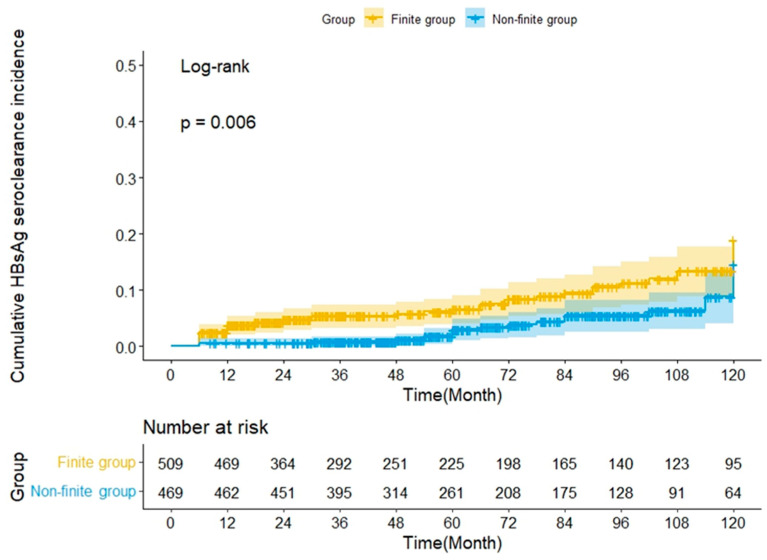
The cumulative HBsAg seroclearance rate over time.

During the study period, 68 patients, including 46 and 22 patients in the finite and non-finite groups, respectively, achieved HBsAg seroclearance. The 5-and 10-year cumulative rates of HBsAg seroclearance were 6.6% and 18.9%, respectively, in the finite group and 3% and 14.6%, respectively, in the non-finite group. The cumulative HBsAg seroclearance rate was significantly higher in the finite group than in the non-finite group (*p* = 0.006, log-rank test; Figure 2).

### 3.3. Predictors of HBsAg Seroclearance

Table 2 shows the risk regression analysis of patients with and without HBsAg seroclearance. In the univariate analysis, TAF therapy, NUC therapy duration, EOT HBsAg status, and relapse times after EOT were significantly different between the patients with and without HBsAg seroclearance. In the multivariate analysis, TAF therapy, duration of NUC therapy, and EOT HBsAg status were found to have a significant association with HBsAg seroclearance.

**Figure 3:**
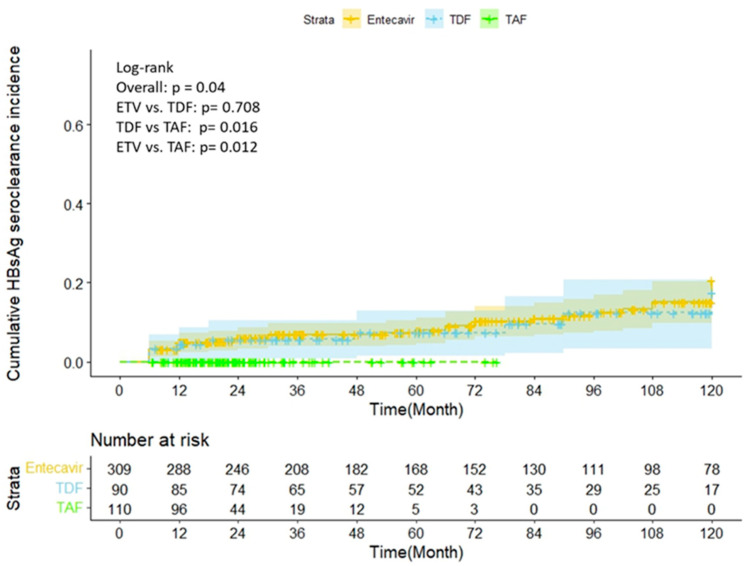
Comparison of the HBsAg seroclearance incidence between different anti-viral therapies.

In the finite group, the HBsAg seroclearance incidence was significantly higher in patients treated with entecavir or TDF than in those treated with TAF. (Entecavir vs. TAF, *p* = 0.012; TDF vs. TAF, *p* = 0.016; log-rank test; Figure 3).

The duration of follow-up was longer in patients treated with entecavir (71 [27.6–120] months) and TDF (68.2 [31.9–116] months) than in those treated with TAF (21.4 [14.7–30.4] months) (entecavir vs. TAF, *p* < 0.001; TDF vs. TAF, *p* < 0.001). Of note, 25 of the 110 patients were treated with entecavir or TDF before switching to TAF. There was no statistically significant difference in the incidence of HBsAg seroclearance between patients who switched or did not switch NUCs (*p* = 0.085). Those with a lower EOT HBsAg had a greater incidence of HBsAg incidence.

**Figure 4:**
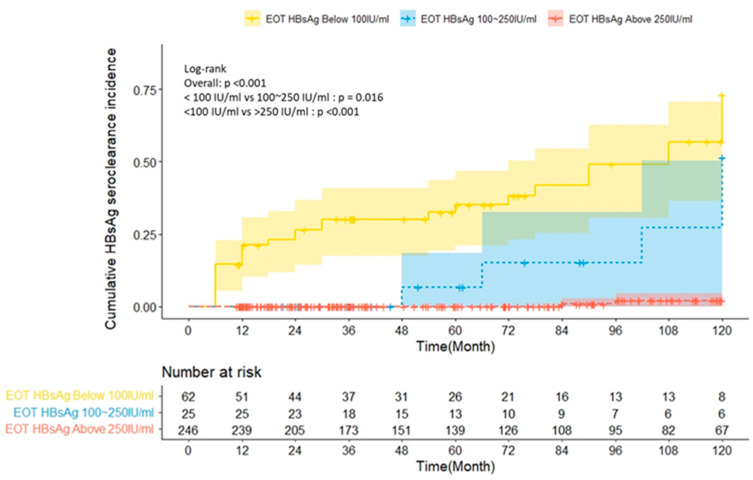
Comparison of the HBsAg seroclearance incidence based on three EOT HBsAg categories (<100, 100–250, and >250 IU/mL).

The HBsAg seroclearance incidence was higher in patients with lower EOT HBsAg levels (*p* < 0.001). Finally, the comparison of HBsAg seroclearance incidence based on three EOT HBsAg categories (<100, 100–250, and >250 IU/mL) revealed that the HBsAg seroclearance incidence was significantly higher in patients with EOT HBsAg levels of <100 IU/mL than in those with higher EOT HBsAg levels (*p* < 0.001; log-rank test; Figure 4).

### 3.4. Safety Evaluation and Incidence of HCC of Finite Therapy

CR occurred in 74 of the 509 patients in the finite group. The median time to CR following EOT was 13.8 (8.5–21.2) months. The cumulative 3-year CR rate was 16.8%. Decompensation occurred in 3.9% of the patients in the finite group, with an incidence rate of 4.1/1000 person-years. In the finite group, two deaths occurred during the study period (0.4%). In one case, death was due to an HBV infection flare-up where retreatment with TDF failed. In the second case, death was due to a pneumonia-related respiratory failure.

During the study period, nine patients developed HCC after NUC therapy, with an HCC incidence rate of 0.41/1000 person-years, while two patients were diagnosed after discontinuing NUC therapy. The durations of HCC diagnosis after EOT were 7.8 and 52.1 months. The rates of HCC did not significantly differ between the finite and non-finite groups (*p* = 0.941). None of the patients who developed HCC had achieved HBsAg seroclearance at the time of HCC diagnosis.

## 4. Discussion

Functional cure is the ultimate objective of HBV infection treatment. HBsAg is a clinically available biomarker of functional cure, whereas hepatitis B core-related antigen (HBcrAg) and HBV RNA are not clinically available [5,15,16]. Importantly, patients who achieve HBsAg seroclearance exhibit better prognosis and are at a lower risk for HCC [17,18]. A few studies that evaluated the incidence of HBsAg seroclearance after NUC therapy discontinuation reported 4-, 5-, and 6-year cumulative incidence rates of 13%, 13–39%, and 13–54.9%, respectively, in cohort sizes ranging from 33 to 1546 patients, with follow-up durations ranging from 18.4 to 66.8 months [8,9,10,19,20,21,22,23,24]. In the present study with a relatively large patient population managed under a uniform treatment policy in a single-center, the cumulative incidence rate of HBsAg seroclearance was relatively higher in the finite group than in the non-finite group.

The incidence of HBsAg seroclearance was relatively low in the present study. Previous studies reported that HBsAg seroclearance increased over time [9,25]. In addition, regular monitoring and periodic visits in clinical trial settings might aid in the identification of a larger number of patients achieving HBsAg seroclearance. In the RETRACT-B study, the patients were evaluated at a median of six visits during a median follow-up period of 18.4 months, with an average gap of 2.8 months between the visits [8]. In the present study, the patients underwent HBsAg testing for a median of six times during the 59.7-month follow-up period, with a median interval of 8.5 months between the HBsAg tests. Additionally, distinct projected EOT HBsAg values for seroclearance were found, and the incidence of HBsAg seroclearance was higher in Western cohorts than in Asian cohorts [25,26]. In the present study, the median EOT HBsAg level was log 2.9 IU/mL and we also included HBeAg-positive patients in the study. This might explain the lower HBsAg seroclearance incidence found in the present study compared to that reported in previous studies.

In agreement with previous studies, we found that the incidence of HBsAg seroclearance was higher in patients with EOT HBsAg levels of <100 IU/mL, those without CR, and those who discontinued NUC therapy [5,8,9,10,11,12,23,24,27,28]. It is important to determine the optimal duration of NUC therapy to achieve a balance among HBsAg seroclearance, NUC side effects and cost, and risk of hepatitis flare-ups after NUC therapy discontinuation. In the present study, NUC therapy lasting more than three years was associated with a significantly lower incidence of HBsAg seroclearance. The exact mechanism underlying the increased HBsAg seroclearance incidence observed after NUC therapy discontinuation remains unclear. One potential mechanism involves the rebound of HBV DNA and HBcrAg, which were reported to be associated with the induction of plasma levels of tumor necrosis factor alpha, interleukins 10 and 12p70, and C-X-C motif chemokine ligand 10 [26]. Establishing an immunological environment that restores HBV-specific T cells leads to HBsAg seroclearance [29,30]. Although HBcrAg and HBV RNA have been demonstrated to predict functional cure of CHB following drug discontinuation, they are not yet clinically available [31].

In the present study, patients who experienced CR with NUC retreatment were less likely to achieve HBsAg seroclearance. CR represents poor HBV control, and patients who are prone to CR are less likely to achieve immunological suppression of covalently closed circular DNA activity, which hampers HBsAg seroclearance. In the present study, the real-world data were subject to insurance reimbursement restrictions. Therefore, the serum HBV DNA levels were measured only in patients with at least a 2-fold increase in ALT levels over the upper normal limit. As a result, we did not compare the incidence of viral recurrence (VR) and CR in HBsAg seroclearance. Furthermore, real-world monitoring of HBV DNA levels might lead to the underestimation of CR rates compared to clinical trial settings [8,9,23,32,33,34].

The STOP-NUC trial demonstrated that the use of entecavir and TDF did not lead to significantly different HBsAg seroclearance incidence rates. However, in RETRACT-B, the incidence of HBsAg seroclearance was higher with TDF than with entecavir [8,12]. Chen et al. reported higher rates of HBsAg seroclearance seven years after TDF discontinuation compared to entecavir discontinuation after at least four years of antiviral therapy (35.4% vs. 22.6%) [35]. In the present study, the HBsAg seroclearance incidence, which was not different between the patients treated with TDF and those treated with entecavir, was however lower in those treated with TAF. Nonetheless, TAF therapy was added to the Taiwanese National Health Insurance in 2019 and the follow-up period was shorter for patients treated with TAF, which might have contributed to the reduced HBsAg seroclearance incidence found in the present study. Furthermore, 70 patients in the current study cohort had previously received entecavir or TDF before switching to TAF. The incidence of HBsAg seroclearance was not significantly different between the patients who received TAF as the only therapy and those who switched from another NUC to TAF. These findings suggest that the observed differences in the incidence of HBsAg seroclearance based on NUC therapy might be impacted by the follow-up duration rather than by the specific NUC.

The incidence of liver decompensation after EOT was higher than that reported in a previous study, although the incidence of mortality related to hepatitis flare-ups was comparable [36]. Timely retreatment may have prevented the flare-related death. The lower rates of CR observed in the present cohort might have contributed to a delay in the diagnosis of a hepatitis flare-up, resulting in the increased incidence of liver decompensation. Additionally, the HCC incidence was not different between the finite and non-finite groups and none of the patients who developed HCC achieved HBsAg seroclearance. These findings indicate that HBsAg seroclearance should be considered an important factor in preventing the development of HCC in patients with CHB. In addition, finite therapy was deemed safe if close monitoring after NUC therapy discontinuation and prompt retreatment in patients with CR could be achieved.

The present study had several limitations. First, the regulations of the Taiwanese National Health Insurance precluded the evaluation of VR and the lack of regular monitoring of blood HBV DNA levels might have contributed to the lower rates of CR and the higher rates of liver decompensation observed following hepatitis flare-ups in clinical practice. Second, due to the restrictions imposed by the Taiwanese National Health Insurance reimbursement criteria, the study patients had fewer HBsAg monitoring datapoints and fewer follow-up visits compared to previous studies. This limitation restricted the frequency of testing and irregular HBsAg monitoring might have contributed to the underestimation of HBsAg seroclearance rates in the present study compared to previous studies. Third, TAF was added to the Taiwanese National Health Insurance after other NUCs, so the patients receiving TAF were followed for a shorter duration. The shorter follow-up duration may have hindered the capacity to detect HBsAg seroclearance. In the end, only two patients who were using TAF in our study achieved HBsAg seroclearance. The limited number of observations could also potentially have led to statistical bias. Future studies with longer follow-up durations are warranted to further elucidate the potential effect of TAF on HBsAg seroclearance. Fourth, our study excluded patients with HCC, cirrhosis, alcoholic liver disease, hepatitis C virus infection, autoimmune hepatitis, or those who had undergone immunosuppressive treatment before initiating NUC therapy. We also excluded patients who were not followed up for at least one year, which may have introduced a possible selection bias into our study. Last, although HBcrAg has been shown to predict the functional cure of CHB following drug discontinuation [37], it is not yet widely available clinically due to the challenges in obtaining the HBcrAg assay in Taiwan. This was a retrospective, real-world analysis that evaluated the outcomes of patients with CHB in the actual clinical context while taking into consideration various health insurance constraints and laws.

## 5. Conclusions

In the present study, the likelihood of achieving HBsAg seroclearance was higher in patients with CHB who discontinued NUC therapy after three years than in those who continued treatment. Those with EOT HBsAg levels of <100 IU/mL and those without CR were more likely to achieve HBsAg seroclearance. In addition, vigilant monitoring and early retreatment after therapy discontinuation were associated with a reduced incidence of severe hepatic decompensation and deaths related to hepatitis flare-ups. Discontinuation of NUC therapy might improve immune surveillance and ease HBV elimination from infected hepatocytes, thereby contributing to the achievement of HBsAg seroclearance and the potential cure of CHB.

## Figures and Tables

**Table 1:** Baseline clinical characteristics of the study population at the start of therapy.

	All (*n* = 978)	Finite Group (*n* = 509)	Non-Finite Group (*n* = 469)	*p* Value
Age (years), mean ± SD	48.1 ± 13	49.1 ± 13.3	46.9 ± 12.5	0.006
Sex (male), *n* (%)	703 (71.9)	374 (73.5)	329 (70.1)	0.247
BMI (kg/m^2^), mean ± SD	24.4 ± 4.8	24.4 ± 4.7	24.5 ± 4.8	0.795
Baseline ALT, U/L, median (IQR) ^†^	148 (79–605.3)	136.5 (78.3–347)	140 (79–412.8)	0.414
Total bilirubin, mg/dL, median (IQR) ^‡^	1.2 (0.8–2.5)	1.3 (0.8–3.2)	1.1 (0.7–2.1)	0.063
HBeAg-negative, *n* (%) ^§^	548 (61.8)	283 (67.5)	265 (56.6)	0.013
HBsAg, log10 IU/mL, median (IQR) ^¶^	3.3 (2.8–3.9)	3.2 (2.6–3.8)	3.3 (2.9–4)	0.016
EOT HBsAg in finite group, and HBsAg of 3 years after treatment in the non-finite group, log10 IU/mL, median (IQR) ^††^	2.9 (2.4–3.3)	2.9 (2.4–3.3)	3.0 (2.5–3.4)	0.452
Baseline hepatitis B DNA, log IU/mL, median (IQR) ^‡‡^	6.6 (5.0–7.6)	6.5 (5.0–7.5)	6.6 (5.0–7.6)	0.66
NUC type: ETV/TDF/TAF, *n* (%)	535 (54.7)/173 (17.7)/270 (27.6)	309 (60.7)/90 (17.7)/110 (21.6)	226 (48.2)/83 (17.7)/160 (34.1)	–
Follow-up duration, months, median (IQR)	59.7 (32.9–101.3)	45.4 (21.1–104.1)	63.4 (42–99.3)	<0.001
NUC therapy duration, months, median (IQR)	36.5 (34.8–36.5)	35.7 (24.1–36.5)	46.3 (38.5–70.1)	<0.001
Number of HBsAg datapoints, median (IQR)	6 (3–9)	4 (2–8)	7 (4.5–10)	<0.001
Interval between HBsAg tests, months, median (IQR)	8.5 (6.5–12)	9 (6–13.2)	8.3 (6.8–12)	0.226
10-year cumulative HBsAg seroclearance incidence	-	18.9%	14.6%	0.006

^†^ Data missing for 103 and 25 patients in the finite and non-finite groups, respectively. ^‡^ Data missing for 157 and 123 patients in the finite and non-finite groups, respectively. ^§^ Data missing for 90 and 1 patient in the finite and non-finite groups, respectively. ^¶^ Data missing for 162 and 130 patients in the finite and non-finite groups, respectively. HbsAg was >250 IU/mL in 85 and 72 patients in the finite and non-finite groups, respectively. ^††^ Data on EOT HBsAg were missing for 176 and 139 patients in the finite and non-finite groups, respectively. EOT HBsAg was >250 IU/mL in 9 and 3 patients in the finite and non-finite groups, respectively. ^‡‡^ Data missing for 87 patients in the finite group. ALT, alanine aminotransferase; BMI, body mass index; ETV, entecavir; HBeAg, HBV e antigen; HBsAg, hepatitis B surface antigen; IQR, interquartile range; NUC, NUC, nucleos(t)ide analog; SD, standard deviation; TAF, tenofovir alafenamide; TDF, tenofovir disoproxil fumarate.

**Table 2:** Risks regression models including patients with and without HBsAg seroclearance.

Variable	HBsAg Seroclearance	No HBsAg Seroclearance	Univariate Analysis		Multivariate Analysis	
	*n* = 68	*n* = 910	HR (95% CI)	*p* Value	HR (95% CI)	*p* Value
Age (years), mean ± SD	48.2 ± 13.9	48 ± 12.9	1 (0.98–1.02)	0.928		
Sex (male), *n* (%)	46 (67.6)	657 (72.2)	1.24 (0.73–2.11)	0.427		
BMI (kg/m^2^), mean ± SD	24.3 ± 4.1	24.5 ± 4.8	0.99 (0.94–1.05)	0.807		
HBeAg-negative, *n* (%) ^†^	41 (60.3)	507 (55.7)	1.4 (0.81–2.42)	0.22		
NUC type						
ETV, *n* (%) (reference)	50 (73.5)	485 (53.3)	Reference			
TDF, *n* (%)	16 (23.5)	157 (17.3)	0.99 (0.55–1.79)	0.191		
TAF, *n* (%) ^‡^	2 (2.9)	268 (29.5)	0.07 (0.02–0.3)	<0.001	0.08 (0.01–0.59)	0.013
Total duration of NUC therapy (months), median (IQR)	36.5 (24.1–36.5)	36.5 (35.8–36.5)	0.97 (0.95–0.99)	0.004	0.95 (0.92–0.99)	0.014
≤36 months, *n* (reference)	28	233	Reference	-		
>36 months, *n*	40	677	0.49 (0.3–0.82)	0.007		
EOT HBsAg, log_10_ IU/mL (IQR) ^§^	1 (−1.2–2.1)	2.9 (2.6–3.3)	0.22 (0.15–0.31)	<0.001	0.2 (0.13–0.32)	<0.001
Relapse Time after EOT in the finite group						
0, *n* (reference)	44	391	Reference	-		
1, *n*	2	72	0.29 (0.07–1.22)	0.041		

^†^ Data missing for 8 and 83 patients in patients with and without HBsAg seroclearance, respectively. ^‡^ Treatment was switched from TDF or entecavir to TAF in 70 patients, including 2 and 68 patients with and without HBsAg seroclearance, respectively. § Data missing for 13 and 260 patients with and without HBsAg seroclearance, respectively. EOT HBsAg was >250 IU/mL in 12 patients without HBsAg seroclearance. BMI, body mass index; CI, confidence interval; ETV, entecavir; HBsAg, hepatitis B surface antigen; HBsAg, hepatitis B surface antigen; HR, hazard ratio; IQR, interquartile range; NUC, NUC, nucleos(t)ide analog; SD, standard deviation; TAF, tenofovir alafenamide; TDF, tenofovir disoproxil fumarate.

## Data Availability

The analyzed data are available on reasonable request.
